# Conditional Inactivation of *Pten* with *EGFR* Overexpression in Schwann Cells Models Sporadic MPNST

**DOI:** 10.1155/2012/620834

**Published:** 2012-12-18

**Authors:** Vincent W. Keng, Adrienne L. Watson, Eric P. Rahrmann, Hua Li, Barbara R. Tschida, Branden S. Moriarity, Kwangmin Choi, Tilat A. Rizvi, Margaret H. Collins, Margaret R. Wallace, Nancy Ratner, David A. Largaespada

**Affiliations:** ^1^Masonic Cancer Center, University of Minnesota, Minneapolis, MN 55455, USA; ^2^Department of Genetics, Cell Biology and Development, University of Minnesota, Minneapolis, MN 55455, USA; ^3^Center for Genome Engineering, University of Minnesota, Minneapolis, MN 55455, USA; ^4^Brain Tumor Program, University of Minnesota, Minneapolis, MN 55455, USA; ^5^Department of Applied Biology and Chemical Technology, The Hong Kong Polytechnic University, Hung Hom, Kowloon, Hong Kong; ^6^Department of Molecular Genetics and Microbiology, University of Florida, Gainesville, FL 32610, USA; ^7^Division of Experimental Hematology and Cancer Biology, Cincinnati Children's Hospital Research Foundation, Cincinnati Children's Hospital Medical Center, Cincinnati, OH 45229, USA; ^8^Division of Pathology and Laboratory Medicine, Cincinnati Children's Hospital Research Foundation, Cincinnati Children's Hospital Medical Center, Cincinnati, OH 45229, USA; ^9^UF Shands Cancer Center, Genetics Institute, University of Florida, Gainesville, FL 32610, USA; ^10^Department of Pediatrics, University of Minnesota, Minneapolis, MN 55455, USA

## Abstract

The genetic mechanisms involved in the transformation from a benign neurofibroma to a malignant sarcoma in patients with neurofibromatosis-type-1- (NF1-)associated or sporadic malignant peripheral nerve sheath tumors (MPNSTs) remain unclear. It is hypothesized that many genetic changes are involved in transformation. Recently, it has been shown that both *phosphatase and tensin homolog* (*PTEN*) and *epidermal growth factor receptor* (*EGFR*) play important roles in the initiation of peripheral nerve sheath tumors (PNSTs). In human MPNSTs, *PTEN* expression is often reduced, while *EGFR* expression is often induced. We tested if these two genes cooperate in the evolution of PNSTs. Transgenic mice were generated carrying conditional floxed alleles of *Pten*, and *EGFR* was expressed under the control of the 2′,3′-*cyclic nucleotide* 3′*phosphodiesterase* (*Cnp*) promoter and a *desert hedgehog* (*Dhh*) regulatory element driving Cre recombinase transgenic mice (*Dhh*-Cre). Complete loss of *Pten* and *EGFR* overexpression in Schwann cells led to the development of high-grade PNSTs. *In vitro* experiments using immortalized human Schwann cells demonstrated that loss of *PTEN* and overexpression of *EGFR* cooperate to increase cellular proliferation and anchorage-independent colony formation. This mouse model can rapidly recapitulate PNST onset and progression to high-grade PNSTs, as seen in sporadic MPNST patients.

## 1. Introduction

Malignant peripheral nerve sheath tumors (MPNSTs) are a rare aggressive form of sarcoma associated with poor prognosis. MPNSTs can occur sporadically or in the context of the autosomal dominant neurofibromatosis type 1 (NF1) tumor syndrome [1–3]. Genomic abnormalities, other than those at the *neurofibromin 1* (*NF1*) locus, have rarely been detected in benign neurofibromas but are numerous in MPNSTs suggesting that many secondary genetic changes are required for the transformation from a benign neurofibroma to MPNST. Recurrent homozygous loss of the *cyclin-dependent kinase inhibitor 2A* (*CDKN2A*) locus was detected in majority of atypical neurofibromas [4]. Mutations in *rat sarcoma viral oncogene homolog* (*RAS*) or its pathway-associated genes may also cause tumor initiation in sporadic MPNSTs that lack *NF1* mutations. Activating mutations in *RAS* (*NRAS* or *KRAS*) or *v-raf murine sarcoma viral oncogene homolog B1* (*BRAF*) mutations have been reported in sporadic MPNSTs [5, 6]. As the MPNST genome contain many abnormalities, it is likely that many other genes drive formation of MPNST. Currently, many of the genetic alterations associated with MPNST initiation/progression are unknown, and identification of these genetic changes may have profound clinical benefits. 

*Phosphatase and tensin homolog* (*PTEN*), a negative regulator of the *PI3 K*/*AKT*/*mTOR* pathway involved in regulation of cell growth and survival, is the most frequently inactivated tumor suppressor gene in sporadic cancer [7]. *Pten* dosage has been shown to be essential for neurofibroma development and malignant transformation in the context of *Kras* activation [8]. We have previously demonstrated the synergistic role of *Pten* inactivation to plexiform neurofibroma tumorigenesis and progression to high-grade PNSTs in the context of *neurofibromatosis 1* (*Nf1*) loss in Schwann cells and/or their precursor cells [9]. The role of *PTEN* in sporadic MPNSTs remains to be elucidated and importantly, there are no rapid animal models currently available that can recapitulate sporadic MPNST. Many cases of NF1-associated and sporadic MPNSTs have demonstrated elevated *epidermal growth factor receptor* (*EGFR*) expression and gene amplification, suggesting that *EGFR* pathway activation may be involved in MPNST tumorigenesis [10–12]. This observation is consistent with the findings of gain- and loss-of-function experiments in *EGFR* transgenic mice [13]. However, the relationship between *PTEN* and *EGFR* in MPNST initiation/progression remains elusive.

In NF1 patients, Schwann cells are believed to be the primary pathogenic cell source in neurofibromas because they show biallelic *NF1* gene mutations [14–16]. *Pten*-regulated pathways have been shown to be major tumor suppressive barriers to PNST progression in Schwann cells in the context of *Nf1* loss [9]. Although normal Schwann cells do not express *Egfr*, Schwann cell precursors and subpopulations of mutant *Nf1* Schwann cells do express *Egfr* [13]. The relationship between *Pten* and *Egfr* in neurofibroma development and progression to aggressive PNST has not been elucidated or modeled in genetically engineered mice. We hypothesized that somatic *Pten* inactivation and *EGFR* overexpression in Schwann cells would promote progressive low-grade to high-grade PNST formation. To test this hypothesis, *Dhh*-Cre was used to elicit recombination of *Pten* ^*flox*/*flox*^ [17] alleles, allowing for inactivation of *Pten* genes in Schwann cells. *EGFR*, under the control of the *Cnp* promoter (*Cnp*-*EGFR*), was used to allow for its overexpression in Schwann cells [13]. *Pten* loss in the context of *EGFR* overexpression in Schwann cells resulted in the formation of high-grade PNSTs. Furthermore, this cooperation was recapitulated in immortalized human Schwann cells *in vitro*.

## 2. Materials and Methods

### 2.1. Generation of Transgenic Animals

**Figure 1:**
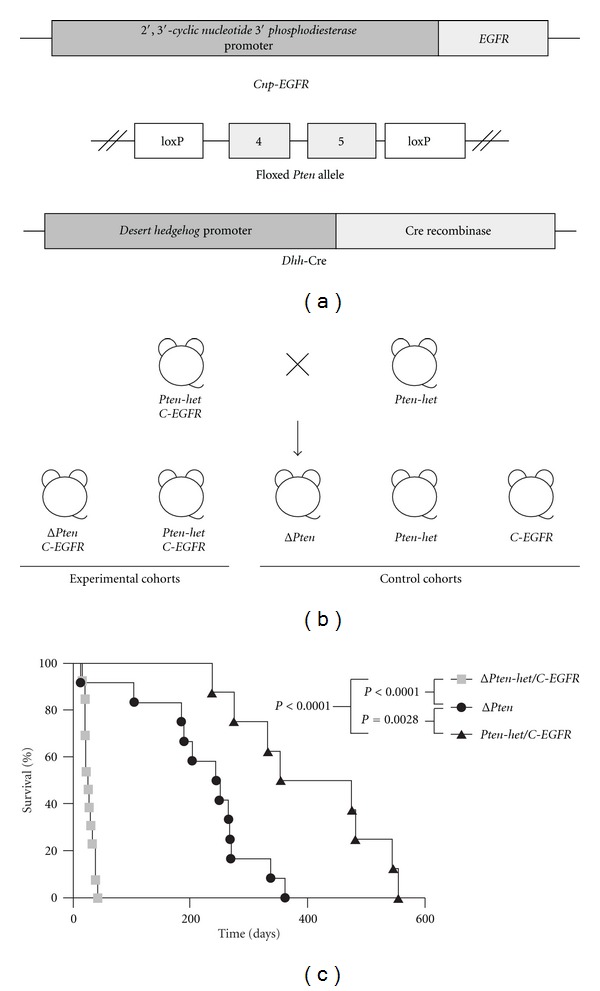
Establishing a novel sporadic peripheral nerve sheath tumor (PNST) progression mouse model. (a) Transgenes used to establish the novel sporadic PNST mouse model. *Cnp*-*EGFR* consists of the *Cnp* regulatory elements driving the *EGFR* gene expression specifically in Schwann cells and/or their precursor cells. Floxed *Pten* allele consists of the essential exons 4 and 5 of the *Pten* gene floxed with loxP sites. *Dhh*-Cre consists of the *Dhh* regulatory elements driving Cre recombinase to remove the loxP sites and allow for the inactivation of the floxed *Pten* alleles specifically in Schwann cells and/or their precursor cells. (b) Breeding strategy for generating experimental and control animals. Transgenic mice each carrying a single transgene was bred to obtain doubly transgenic mice *Dhh*-Cre; *Pten* ^*flox*/+^ mice (*Pten*-*het*). Doubly transgenic mice were then bred with remaining transgene to obtain triple transgenic *Dhh*-Cre; *Pten* ^*flox*/+^; *Cnp*-*EGFR* mice (*Pten*-*het*/*C*-*EGFR*). Finally, *Pten*-*het* mice were bred with *Pten*-*het*/*C*-*EGFR* mice to obtain the required experimental and control cohorts. *Dhh*-Cre; *Pten* ^*flox*/*flox*^; *Cnp*-*EGFR* (Δ*Pten*/*C*-*EGFR*) and *Dhh*-Cre; *Pten* ^*flox*/+^; *Cnp*-*EGFR* (*Pten*-*het*/*C*-*EGFR*) experimental cohorts. *Dhh*-Cre; *Pten* ^*flox*/*flox*^ (Δ*Pten*), *Pten*-*het* and *Cnp*-*EGFR* (*C*-*EGFR*) control cohorts. (c) Kaplan-Meier survival curves of various experimental and control cohorts. *Pten* dosage augmented the peripheral nervous system phenotype in the context of *EGFR* overexpression in Schwann cell and/or their precursor cells, resulting in decreased survival. *P*: log-rank test.

Generation of transgenic mice carrying the *Dhh* gene regulatory element driving Cre recombinase (*Dhh*-Cre) has been previously described [18] (Figure 1(a)). Transgenic mice carrying the floxed *Pten* allele with the essential exons 4 and 5 of the *Pten* gene floxed with loxP sites have been previously described [17] (Figure 1(a)). The transgenic mouse carrying the *Cnp* gene regulatory element driving *EGFR* (*Cnp*-*EGFR*) has been previously described [13]. Breeding strategy for generating various experimental, and control cohorts is shown in Figure 1(b). Animals were sacrificed when moribund due to paralysis and necropsy performed. All animal work was conducted according to the University of Minnesota's approved animal welfare protocol.

### 2.2. PCR Genotyping

Identification of the various genotypes from both adult transgenic animal and pups was performed as follows. Firstly, genomic DNA was isolated from tail clippings using standard proteinase K treatment, phenol-chloroform extraction, and ethanol precipitation. Genomic DNA was then dissolved in sterile TE (10 mM tris-HCl (pH7.5), 1 mM EDTA (pH 8)) and quantified using a Nanodrop spectrophotometer. PCR genotyping was performed using 50 ng of diluted genomic DNA as template in a 25 *μ*L PCR reaction volume. PCR primers used for *Dhh*-Cre were forward 5′-CTGGCCTGGTCTGGACACAGTGCCC-3′ and reverse 5′-CAGGGTCCGCTCGGGCATAC-3′ (amplicon 385 bp); *Pten* floxed allele were forward 5′-AAAAGTTCCCCTGCTGATTTGT-3′ and reverse 5′-TGTTTTTGACCAATTAAAGTAGGCTGT-3′ (WT amplicon 310 bp and floxed allele amplicon 435 bp); *Cnp*-*EGFR* were forward 5′-TGACATCTCCTCCTCCCTTC-3′ and reverse 5′-TGCCCAACTGCGTGAGC-3′ (amplicon 380 bp). PCR conditions for GoTaq Green Master Mix (Promega) were used according to the manufacturer's instructions with an initial denaturing step of 95°C for 2 min; 30 or 35 cycles of denaturing at 95°C for 25 sec, annealing at 55°C for 35 sec and extension at 72°C for 65 sec; followed by a final extension at 72°C for 5 min. PCR products were separated on a 2% agarose gel and genotype determined by the absence or presence of expected amplicons.

### 2.3. Peripheral Nerve Tumor Analysis

PNSTs were carefully removed from the sacrificed animals under a dissecting microscope (Leica), washed, and placed in cold phosphate buffered saline (PBS). Any abnormal sciatic nerves, brachial plexi, and/or sacral plexi were also removed. Trigeminal nerves attached to the brain were also observed for any abnormalities and removed. The number of enlarged dorsal root ganglia was counted for the whole spinal cord. All reasonably sized tumor nodules (>1 mm in diameter) were carefully removed using fine forceps and placed in fresh cold PBS.

### 2.4. Hematoxylin-Eosin (HE) Staining

Sections for histology were only taken from larger tumor nodules (>1 mm in diameter). Tissues were fixed in 10% formalin, routinely processed, and embedded in paraffin. Sections for histology were cut at 5 microns from the paraffin blocks using a standard microtome (Leica), mounted and heat-fixed onto glass slides. Slides were either stained with HE using standard protocols, immunohistochemistry, and/or toluidine blue staining as described in the next section.

### 2.5. Immunohistochemistry (IHC) and Toluidine Blue (TB) Staining

Formalin fixed-paraffin embedded sections from various tissues were sectioned at 5 microns, mounted and heat-fixed onto glass slides to be used for IHC analyses. Briefly, the glass section slides were dewaxed and rehydrated through a gradual decrease in ethanol concentration. The antigen epitopes on the tissue sections were then unmasked using a commercially available unmasking solution (Vector Laboratories) according to the manufacturer's instructions. The tissue section slides were then treated with 3% hydrogen peroxide to remove any endogenous peroxidases. Blocking was performed at 4°C using a M.O.M. mouse immunoglobulin-blocking reagent (Vector Laboratories) or in appropriate normal serum from the host of the secondary antibody (5% serum in PBS) in a humidified chamber for several hours. For IHC, sections were then incubated overnight at 4°C in a humidified chamber using various primary antibodies at the indicated dilutions: Ki67 (1 : 200) (Novocastra), S100ß (1 : 100) (Santa Cruz), Pten (1 : 200) (Cell Signaling), phospho-Erk1/2 (1 : 400) (Cell Signaling), phospho-Akt (Ser473) (D9E) (1 : 250) (Cell Signaling), and phospho-S6 (Ser240/244) (1 : 200) (Cell Signaling). After primary incubation, sections were washed thoroughly in PBS before incubating with horseradish peroxidase-secondary antibody raised against the primary antibody initially used. After thorough washes with PBS, the sections were treated with freshly prepared DAB substrate (Vector Laboratories) and allowed for adequate signal to develop before stopping the reaction in water. Finally, sections were then lightly counter stained with hematoxylin, dehydrated through gradual increase in ethanol concentration, cleared in xylene, and mounted in Permount (Fisher).

TB staining for mast cells were performed using standard protocols. Briefly, sections were dewaxed and rehydrated to water, stained with toluidine blue working solution (0.1% toluidine blue O in 0.9% sodium chloride pH 2.3) for 2-3 min, washed 3 times with distilled water before dehydrating quickly through a series of alcohols, clearing in xylene and finally mounted in Permount.

### 2.6. Histologic Evaluation

Sections stained with HE; antibodies to Ki67 and S100ß antigens; and with toluidine blue were evaluated for all tumors [19]. Each sample was graded using established criteria for tumors arising in genetically engineered mice [20, 21]. Briefly, low-grade PNSTs exhibited low cellularity with little if any nuclear atypia and mitotic activity. High-grade PNSTs were increasingly cellular with increasing nuclear atypia and increasing mitotic activity.

### 2.7. Microarray Gene Expression Analysis

Differentially expressed genes were defined as genes with expression levels at least 3-fold higher or lower in target groups compared to normal Schwann cells (N-SC) after applying Benjamini and Hochberg false discovery rate correction [22] (*P* ≤ 0.05) in GeneSpring GX v7.3.1. Custom Affymetrix chip definition file [23] based on RefSeq target definitions (Hs133P REFSEQ Version 8) was downloaded and used to provide accurate interpretation of GeneChip data. Heatmaps were generated using GeneSpring GX v7.3.1.

### 2.8. Gene Knockdown and Overexpression

Cultured normal Schwann cells were derived from a healthy individual's sciatic nerve [24] and immortalized by overexpressing both human *telomerase reverse transcriptase *(*TERT*) and mouse *cyclin-dependent kinase 4* (*Cdk4*) to allow for *in vitro *studies-immortalized human Schwann cells referred to as HSC2*λ* hereafter (H. Li & M. R. Wallace, manuscript in preparation). The plasmid vectors shown in Figure 5(a) along with a plasmid containing *piggyBac 7 *(*PB7*) transposase [25] under the control of a cytomegalovirus promoter (*construct not shown*) were transfected into HSC2*λ* cells using the Neon transfection system, according to manufacturer's protocol (Invitrogen). *PiggyBac* (*PB*) transposon expression constructs contain the *eukaryotic translation elongation factor 1 alpha 1 *(*EEF1A1*) promoter to drive expression of a *PTEN* shRNA and/or *EGFR* cDNA and include a puromycin resistance gene and *green fluorescent protein* (*Gfp*) cDNA for selection purposes. The *PB*-control vector contains the *Luciferase* and *Gfp* reporter genes under the control of the *cytomegalovirus early enhancer element* and *chicken beta-actin* (*CAG*) promoter. Following transfection, cells underwent selection in standard DMEM full media containing 4 *μ*g/mL puromycin (Invitrogen). Transposon-mediated gene transfer was validated by *Gfp* expression and quantitative PCR.

### 2.9. Quantitative PCR in Human Culture Cells

Total RNA was extracted from 1 million cells using the High Pure RNA Isolation Kit (Roche). RNA was quantified by Nanodrop (Thermo Scientific) and its purity confirmed by agarose gel electrophoresis. One microgram of RNA was used to synthesize cDNA using the Transcriptor First Strand Synthesis (Roche) using both random hexamer and oligo dT primers. Quantitative PCR reactions were conducted using LightCycler 480 SYBR I Green (Roche) and run on an Eppendorf Mastercycler ep gradient S. Primers for *PTEN *were forward 5′-TTGGCGGTGTCATAATGTCT-3′ and reverse 5′-GCAGAAAGACTTGAAGGCGTA-3′; *EGFR *were forward 5′-TTCCAAATTCCCAAGGACC-3′ and reverse 5′-GGGCTCTGGAGGAAAAGAAA-3′; *actin beta* (*ACTB*) forward 5′-CACAGGGGAGGTGATAGCAT-3′ and reverse 5′-CTCAAGTTGGGGGACAAAAA-3′. Data were analyzed using RealPlex software, calibrated to *ACTB* levels, normalized to *PB*-control cells and averaged over three experimental replicates.

### 2.10. Proliferation Assays

Proliferation assays were set up in a 96-well format with 100 cells plated per well in DMEM full media containing 4 *μ*g/mL puromycin (Invitrogen). Proliferation was assessed every 24 hours over 6 days by the MTS assay (Promega) according to the manufacturer's protocol. Absorbance was read at 490 nm to determine proliferation and 650 nm to account for cellular debris on a BioTek Synergy Mx automated plate reader. Experiments were conducted with 4 technical replicates and 3 experimental replicates. Data shown is a representative experimental replicate.

### 2.11. Colony Forming Assay

Six-well plates were prepared with bottom agar (3.2% SeaPlaque Agar in DMEM full media) and allowed to solidify before 10,000 cells in top agar (0.8% SeaPlaque Agar in DMEM full media) were plated and allowed to solidify. DMEM full media with 4 *μ*g/mL puromycin (Invitrogen) was plated over the cells and cells were incubated under standard conditions (5% CO_2_ and 37°C) for 2 weeks. Top media was removed and cells were fixed in 10% formalin containing 0.005% Crystal Violet for 1 hour at room temperature. Formalin was removed and colonies were imaged on a Leica S8 AP0 microscope. Twelve images per cell line were taken and automated colony counts were done using ImageJ software (NIH). Results shown are a representative example of 3 independent experiments. 

## 3. Results

### 3.1. Early Postnatal Lethality Results from *Pten* Inactivation and *EGFR* Overexpression in Schwann Cells

Transgenes used to generate the sporadic peripheral nerve sheath tumor progression mouse model are shown in Figure 1(a). Singly transgenic mice each carrying the three individual transgenes (*Dhh*-Cre, *Pten* ^*flox*/+^, and *Cnp*-*EGFR*) were interbred to generate both experimental and control cohorts (Figure 1(b)). A significant difference in survival was observed between experimental cohort *Dhh*-Cre; *Pten* ^*flox*/*flox*^; *Cnp*-*EGFR* (abbreviated as Δ*Pten*/*C*-*EGFR* hereafter) and control cohort *Dhh*-Cre; *Pten* ^*flox*/+^; *Cnp*-*EGFR* (abbreviated as *Pten*-*het*/*C*-*EGFR* hereafter) (*P* < 0.0001, log-rank test) (Figure 1(c)). Biallelic inactivation of *Pten* and *EGFR* overexpression (Δ*Pten*/*C*-*EGFR*) in Schwann cells led to rapid postnatal death, resulting in a median survival age of 26 days (Figure 1(c)). A lesser difference in survival was observed between Δ*Pten*/*C*-*EGFR* and a control cohort *Dhh*-Cre; *Pten* ^*flox*/*flox*^ (abbreviated as Δ*Pten* hereafter) (*P* < 0.0001) (Figure 1(c)). There was also a significant difference in survival rate observed between control cohorts Δ*Pten* and *Pten*-*het*/*C*-*EGFR* (*P* = 0.0028) (Figure 1(c)). The occurrence of peripheral nervous system phenotypes and median survival age for various cohorts are shown in Table 1. Increasing levels of *Pten* with *EGFR* (*Pten-het/C*-*EGFR*) partially alleviated the severe phenotype, leading to an increase in survival and median survival age of 415 days (Figure 1(c)). Biallelic inactivation of *Pten* (Δ*Pten*) alone resulted in a median survival age of 247-days (Figure 1(c)). In contrast, *Dhh*-Cre; *Pten* ^*flox*/+^ (abbreviated as *Pten*-*het* hereafter) control mice displayed no obvious phenotype as previously observed [9], while a similar phenotype was observed in *Cnp*-*EGFR* (abbreviated as *C*-*EGFR* hereafter) control mice as previously described [13].

### 3.2. Severe Peripheral Nervous System Phenotype Observed in Δ*Pten*/*EGFR* Animals

**Figure 2:**
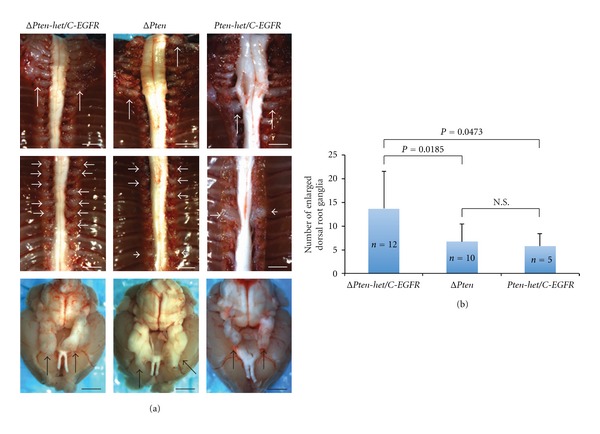
*Pten* dosage with *EGFR* overexpression affected enlarged dorsal root ganglia tumor multiplicity. (a) Left: representative of an early-onset peripheral nervous system phenotype observed in a 38-day *Dhh*-Cre; *Pten* ^*flox*/*flox*^; *Cnp*-*EGFR* (Δ*Pten*/*C*-*EGFR*) experimental mouse. Brachial plexus: majority of the dorsal root ganglia and trigeminal nerves were enlarged. Middle: representative of a late-onset peripheral nervous system phenotype observed in a 104-day *Dhh*-Cre; *Pten* ^*flox*/*flox*^ (Δ*Pten*) control mouse. Brachial plexus: several dorsal root ganglia and trigeminal nerves were enlarged. Right: representative of a late-onset peripheral nervous system phenotype observed in a 274-day *Dhh*-Cre; *Pten* ^*flox*/+^; *Cnp*-*EGFR* (*Pten-het*/*C*-*EGFR*) control mouse. Brachial plexus: several dorsal root ganglia and trigeminal nerves were enlarged. Top panels: brachial plexi; middle panels: dorsal root ganglia; bottom panels: brain with trigeminal nerves; arrows indicate peripheral nervous system phenotype; scale bars, 2 mm. (b) Statistically significant differences in the number of enlarged dorsal root ganglia isolated from Δ*Pten*/*C*-*EGFR* experimental cohort compared with control cohorts (Δ*Pten* and *Pten-het*/*C*-*EGFR*). Mean ± standard deviation; *P*: unpaired *t*-test; *n*: number of mice evaluated in each cohort; N.S.: nonsignificant.

Δ*Pten/C*-*EGFR *experimental animals displayed multiple enlarged dorsal root ganglia and enlarged peripheral nerves: brachial plexi, sacral plexi, trigeminal and sciatic nerves (Figure 2(a), *left* and Table 1). Δ*Pten* control animals displayed a similar peripheral nervous system phenotype but with delayed latency and at a significantly reduced tumor multiplicity (Figure 2(a), *middle* and Table 1). *Pten-het/C*-*EGFR* animals displayed a similar peripheral nervous system phenotype with Δ*Pten* control animals but with a latency of 415 days (Figure 2(a), *right*). The enlargement of peripheral nerves and dorsal root ganglia has been described for *C-EGFR* [13].

Importantly, *Pten* loss contributed to enlarged dorsal root ganglion formation. Δ*Pten/C*-*EGFR* animals had significantly more enlarged dorsal root ganglia (13.7 ± 7.8, mean ± standard deviation), compared with (i) Δ*Pten* animals (*P* = 0.0185, unpaired *t*-test) and (ii) *Pten-het/C*-*EGFR* animals (*P* = 0.0473, unpaired *t*-test) (Figure 2(b) and Table 1). There was no statistical significance in the number of enlarged dorsal root ganglia between Δ*Pten* (6.7 ± 3.8) and *Pten-het/C*-*EGFR* (5.8 ± 2.6) animals (Figure 2(b) and Table 1). Δ*Pten/C*-*EGFR* experimental animals displayed the following peripheral nerve phenotype: enlarged brachial plexi (100%), enlarged trigeminal nerves (92%), enlarged lumbar sacral plexi (50%), and enlarged sciatic nerves (50%) (Table 1). Δ*Pten* and *Pten-het/C*-*EGFR* animals had a similar incidence of enlarged peripheral nerves: enlarged brachial plexi (100%), enlarged trigeminal nerves (90~100%), enlarged lumbar plexi (10~20%), and enlarged sciatic nerves (80%) (Table 1). Histological analysis showed that enlarged peripheral nerves from Δ*Pten* animals were low-grade PNSTs, while enlarged peripheral nerves from *Pten-het/C*-*EGFR* control animals were graded as hyperplasia to low-grade PNSTs based on the same grading criteria (Table 1). *C-EGFR* control animals display only peripheral nerve hyperplasia, with rare incidence of neurofibroma.

### 3.3. Histopathological and Immunohistochemical (IHC) Analyses

**Figure 3:**
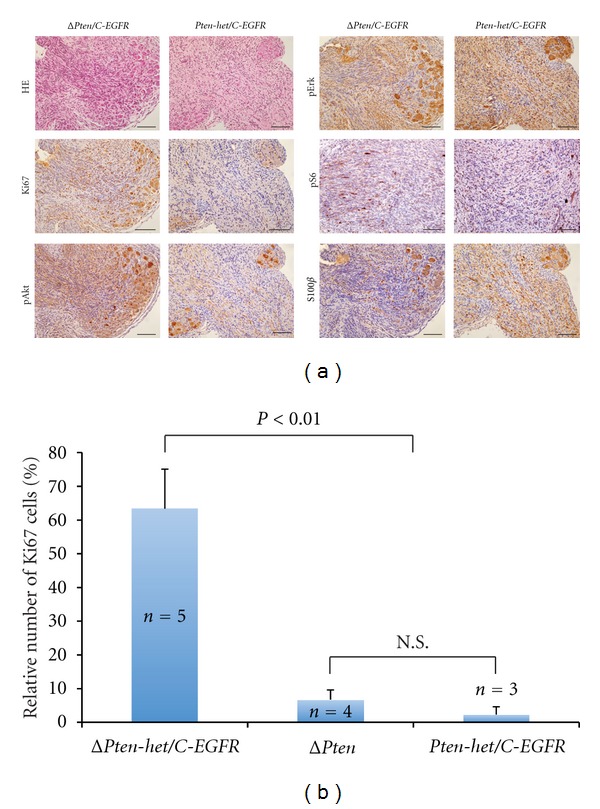
Histological analyses of peripheral nervous system phenotype. Standard hematoxylin-eosin staining (HE) and immunohistochemical (IHC) staining were performed on all peripheral nervous system tissue sections. (a) Representative HE and IHC staining of peripheral nerves taken from a *Dhh*-Cre; *Pten* ^*flox*/*flox*^; *Cnp*-*EGFR* (Δ*Pten*/*C*-*EGFR*) experimental and *Dhh*-Cre; *Pten* ^*flox*/+^; *Cnp*-*EGFR* (*Pten-het*/*C*-*EGFR*) control mice using antibodies against the proliferative marker (Ki67), Schwann cell/oligodendrocyte lineage marker (S100ß), activated *Ras/Mapk/Erk* signaling by phospho-Erk1/2 (pErk), activated *Pi3k/Akt* signaling by phospho-Akt (pAkt) detection, and activated *mTor* signaling by phospho-S6 (pS6). Negative controls, sections incubated without the primary antibody gave no significant signal above background (*data not shown*). (b) Semiquantitative analysis of proliferative peripheral nerve cells in various control and experimental cohorts. Representative peripheral nerves were isolated from each cohort and IHC stained using the Ki67 proliferative marker. The number of Ki67-positive peripheral nerve cells was counted and shown as a percentage of total cells per counted field of view at 20x magnification (mean ± standard deviation). Peripheral nerves were taken from Δ*Pten*/*C*-*EGFR* experimental mice, *Dhh*-Cre; *Pten* ^*flox*/*flox*^ (Δ*Pten*) and *Pten-het*/*C*-*EGFR* control mice. No Ki67-positive cells were detected in sciatic nerves isolated from FVB/N mice (Supplementary Figure 1). *n*, number of mice from each cohort; N.S.: nonsignificance between indicated cohorts; *P*: unpaired *t*-test.

Histopathological and immunohistochemical (IHC) analyses of peripheral nervous system tissues taken from Δ*Pten/C*-*EGFR* experimental animals demonstrated high-grade PNSTs compared with hyperplasia to low-grade PNSTs seen in *Pten*-*het/C*-*EGFR* animals by histology and Ki67 staining criteria as defined [20, 21] (Figures 3(a) and 3(b)). Mast cells were detected in these enlarged peripheral nerves by toluidine blue staining (*data not shown*). Enlarged peripheral nerves taken from Δ*Pten* control animals were low-grade PNSTs as previously observed [9]. Enlarged peripheral nerves taken from representative experimental and control animals were positive for S100ß staining, consistent with nerve association (Figure 3(a)). Enlarged peripheral nerves taken from Δ*Pten/C*-*EGFR* and *Pten*-*het/C*-*EGFR* were Ki67-positive at varying intensities, indicative of cell proliferation (Figure 3(a)). Analyses for proliferation demonstrated significant differences (*P* < 0.01) in the number of Ki67-positive cells in Δ*Pten/C*-*EGFR* animals with high-grade PNSTs when compared with other cohorts with hyperplasia/low-grade to low-grade tumors seen in *Pten*-*het/C*-*EGFR* and Δ*Pten* animals, respectively, (Figure 3(b) and Supplementary Figure 1: available online at doi:10.1155/2012/620834). Importantly, the histological and immunohistochemical features of the rapidly growing tumors are consistent with genetically engineered mouse model (GEMM) high-grade PNST and mimic human sporadic MPNST [20]. In a representative tumor sample, we detected hypercellularity, haphazard cell arrangement, poor cell differentiation, variable nuclear pleomorphism, and high mitotic index (Supplementary Figure 2). In addition, all GEMM-PNSTs showed association between nerves and scattered S100*β*-positive cells (Figure 3(a) and Supplementary Figure 2), typical of GEMM high-grade PNSTs.

Enlarged peripheral nerves taken from both Δ*Pten/C*-*EGFR* and *Pten*-*het/C*-*EGFR* animals were both pErk1/2 positive by IHC at higher levels than detected in normal nerves (Supplementary Figure 1), thus confirming that *EGFR* overexpression in Schwann cells and/or their precursor cells resulted in activated *Ras*/*Mapk/Erk* signaling (Figure 3(a)). The enlarged peripheral nerves taken from Δ*Pten/C*-*EGFR* and Δ*Pten* animals were also pAkt positive by IHC, at levels were higher than detected in normal nerves (Supplementary Figure  1), thus confirming the conditional inactivation of *Pten* in Schwann cells and/or their precursor cells results in activated *Pi3k*/*Akt*/*mTor* signaling (Figure 3(a)). Activation of the *mTor* signaling pathways was evident in both Δ*Pten/C*-*EGFR* and *Pten*-*het/C*-*EGFR* animals by pS6 staining (Figure 3(a)). However, the intensity of pS6 staining was higher in the majority of enlarged peripheral nerves from Δ*Pten/C*-*EGFR* animals.

### 3.4. Microarray Gene Expression Analysis of Human Peripheral Nerve Tumor Samples

**Figure 4:**
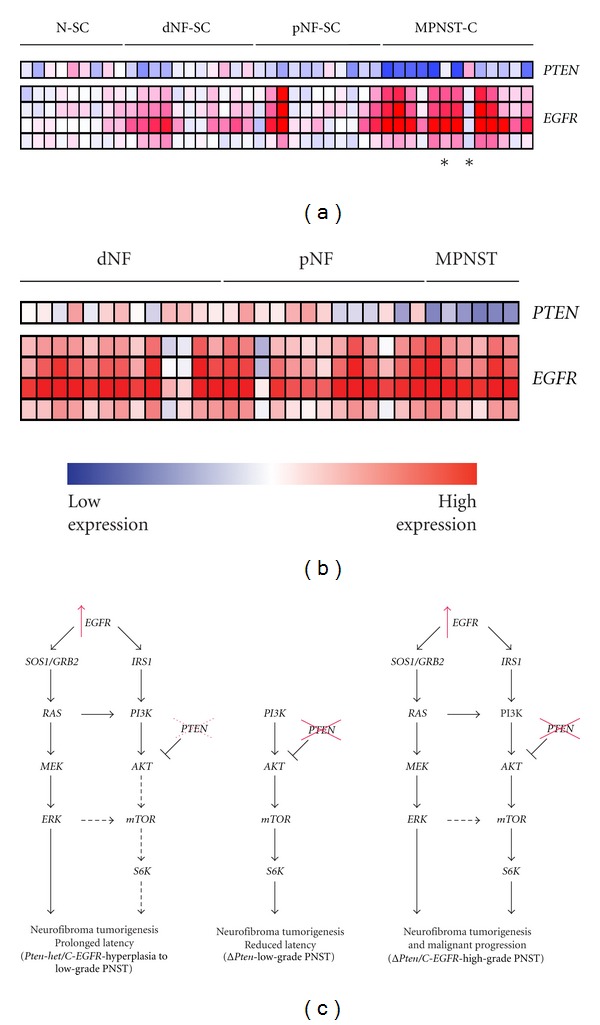
Expression microarray analysis of *PTEN* and *EGFR* in human peripheral nerve tumors. (a) Purified human Schwann cells from normal sciatic nerve (N-SC), dermal neurofibroma cell lines (dNF-SC), and plexiform neurofibroma cell lines (pNF-SC). Transformed cells from malignant peripheral nerve sheath cell lines (MPNST-C). Asterisks indicate sporadic MPNST samples. (b) Solid dermal neurofibromas (dNF), plexiform neurofibromas (pNF), and malignant peripheral nerve sheath tumors (MPNST). Four different probes for EGFR were used. Red, increase in red intensity as expression increases; Blue, increase in blue intensity as expression decreases. (c) Conditional inactivation of *Pten* and *EGFR* overexpression in Schwann cells resulted in high-grade PNST initiation and/or progression due to the upregulation of both *Ras*/*Mapk/Erk* and *Pi3k*/*Akt*/*mTor* signaling pathways (*right*). Inactivation of *Pten* alone resulted in reduced latency with low-grade PNST tumorigenesis at low penetrance (*middle*). Conditional inactivation of *Pten* alone can result in low-grade PNST tumorigenesis via the upregulation of the *Pi3k*/*Akt*/*mTor* signaling pathway. Partial conditional inactivation of *Pten* in the context of *EGFR* overexpression in Schwann cells resulted in prolonged latency with hyperplasia to low-grade PNST tumorigenesis at low penetrance (*left*), resulting in upregulation of *Ras*/*Mapk/Erk* and slight upregulation of the *Pi3k*/*Akt*/*mTor* signaling pathways. *SOS1*: *son of sevenless homolog 1*; *GRB2*: *growth factor receptor-bound protein 2*; *IRS2*: *insulin receptor substrate 2*; *Dhh*-Cre; *Pten* ^*flox*/*flox*^; *EGFR* (Δ*Pten/C*-*EGFR*), *Dhh*-Cre; *Pten* ^*flox*/+^; *EGFR* (*Pten*-*het/C*-*EGFR*) and *Dhh*-Cre; *Pten* ^*flox*/*flox*^ (Δ*Pten*) animals.

Both *PTEN* and *EGFR* levels in purified Schwann cells taken from human peripheral nerve and neurofibroma cells; transformed cells taken from MPNST cell lines (Figure 4(a)) and solid tumors (Figure 4(b)) at various stages of disease were analyzed by microarray gene expression analysis. 

Although there may be a trend towards reduced *PTEN* expression levels at early stages of the disease, there was a dramatic decrease in its expression level in the malignant stage of the disease (Figures 4(a) and 4(b)). *EGFR* mRNA expression increases as disease progresses from a benign to a malignant tumor (Figures 4(a) and 4(b)), likely due to the homogeneity of the cell population. There appears to be a selective pressure to lose *PTEN* and gain *EGFR* expression levels during tumor evolution.

### 3.5. Knockdown of *PTEN* and Overexpression of *EGFR* Cooperate *In Vitro* to Oncogenically Transform Immortalized Human Schwann Cells

**Figure 5:**
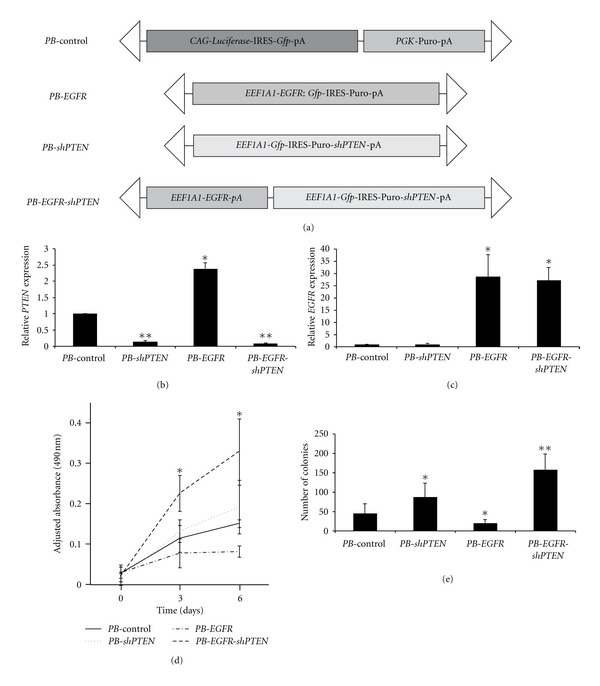
Knockdown of *PTEN* and overexpression of *EGFR* cooperate *in vitro* to oncogenically transform immortalized human Schwann cells. (a) *PiggyBac* (*PB*) constructs used to knock down *PTEN* (*PB*-*shPTEN*) and/or overexpress *EGFR *(*PB*-*EGFR*) in HSC2*λ* immortalized human Schwann cells*. CAG*: cytomegalovirus early enhancer element and chicken beta-actin promoter; *PGK*: *phosphoglycerate kinase*; *EEF1A1*: *eukaryotic translation elongation factor 1 alpha 1 *promoter; IRES: internal ribosome entry site; *Gfp*: *green fluorescent protein*; pA: polyadenylation signal; Puro: puromycin resistance gene; triangles: *PB*-specific inverted terminal repeat sequences. Quantitative PCR analysis demonstrating that *PTEN *mRNA levels are reduced (b) and *EGFR *mRNA levels are increased (c) when these constructs are stably transfected into HSC2*λ* cells. (d) MTS proliferation assay shows that *PTEN *knockdown or *EGFR *overexpression alone do not change the rate of proliferation compared to control transfected cells, but when combined significantly increase cellular proliferation. (e) Soft agar colony formation assay demonstrates that *PTEN *knockdown moderately increases colony formation, but in the context of *EGFR *overexpression, reduction in *PTEN *significantly increases the number of colonies formed. *P < 0.05 and **P < 0.0001, unpaired *t*-test; mean ± standard deviation.

Knockdown of *PTEN* and overexpression of *EGFR* were performed in immortalized human Schwann cells to confirm the phenotype observed in our novel sporadic mouse model. The constructs used to express a *PTEN* shRNA or *EGFR* cDNA alone or in combination are shown in Figure 5(a). HSC2*λ* cells were transfected with various constructs and *piggyBac *transposase to induce transposon-mediated gene transfer of these constructs (Figure 5(a)). Quantitative PCR analysis demonstrated expression of these constructs resulted in the expected expression changes at the mRNA level. *PTEN* mRNA level was reduced by greater than 7-fold when *PTEN* was knocked down alone, and greater than 11-fold when *PTEN* was knocked down in the context of *EGFR* overexpression. *EGFR* was overexpressed by approximately 28-fold either alone or in the context of *PTEN* knockdown (Figures 5(b) and 5(c)). While there was no significant change in the rate of cellular proliferation between cells that expressed the *PTEN* shRNA or *EGFR* alone, there was a significant increase in proliferation when *PTEN* was knocked down in the context of *EGFR* overexpression (*P* < 0.05, unpaired *t*-test) (Figure 5(d)). Similarly, anchorage-independent growth as measured by soft agar colony formation was enhanced by greater than 3.5-fold when *PTEN* shRNA and *EGFR* were expressed in combination (*P* < 0.0001) (Figure 5(e)).

## 4. Discussion

It is becoming increasingly clear that *PTEN*-regulated pathways represent important major tumor suppressive barriers to peripheral nerve sheath tumorigenesis. In the current study, we addressed the hypothesis that *PTEN* cooperates with *EGFR* in the genetic evolution from a benign to malignant PNST. This hypothesis was established in a recent *Sleeping Beauty* transposon insertional mutagenesis forward genetic screen for genes responsible for MPNST, which was performed in the genetic background of *EGFR* over-expressing Schwann cells (manuscript in preparation). There is a fundamental genetic difference in the evolution of sporadic MPNST between human and our mouse model: human sporadic MPNSTs have a prolonged latency due to the accumulation of multiple genetic alterations within one clone, while our transgenic mouse model develops oligoclonal high grade PNSTs rapidly. Thus, our mouse model may not mimic the genetic diversity of the human disease and may reflect only one genetic subtype of MPNST. Nevertheless, our current study provides a rapid animal model for the elucidation of genetic mechanisms that occur in the context of sporadic or *de novo* MPNST progression.

*EGFR* overexpression has been detected in several human cancers such as breast, gut, and hepatocellular carcinoma [26–29]. NF1-associated and sporadic MPNSTs have *EGFR* gene amplifications and overexpression at both the mRNA and protein level, supporting its role in MPNST tumorigenesis [10, 11]. In addition to its possible role in tumor progression, higher tumor *EGFR* expression has been correlated with worse prognosis in patients with MPNST [30]. Activation of *EGFR* triggers signaling processes that promote cell proliferation, migration, adhesion, angiogenesis, and inhibition of apoptosis [31]. A transgenic mouse model with *EGFR* overexpression in Schwann cells elicited features of neurofibromas such as hyperplasia, excess collagen, mast cell accumulation, and progressive dissociation of non-myelin-forming Schwann cells from axons [13]. In these transgenic mice, there was no evidence of high-grade PNSTs suggesting that additional genetic events are required for the evolution from a benign neurofibroma to a MPNST. Analysis of microarray data from cell lines and bulk tumors isolated from human neurofibromas and MPNSTs indicate that there is a selective pressure to lose *PTEN* expression, while *EGFR* expression is induced during disease progression (Figures 4(a) and 4(b)). It has recently been shown that loss of *PTEN* is associated with elevated *EGFR* and *HER2* expression and worse prognosis in salivary gland cancer, further demonstrating the importance of these two genes in other cancer types [32]. In the current study, we have provided genetic evidence that loss of *Pten* cooperates with *EGFR* overexpression in Schwann cells, in the evolution from a low-grade to high-grade PNST using our GEMM. The genetic mechanism for the transformation involves the upregulation of both *Ras*/*Mapk/Erk* and *Pi3k*/*Akt*/*mTor* signaling pathways (Figures 3(a) and 4(c)). It is not surprising that a similar mechanism(s) was also observed in a GEMM with conditional inactivation of *Nf1* and *Pten* in Schwann cells [9]. Further, we show that this mechanism is likely occurring in human patients, as reduction of *PTEN* and overexpression of *EGFR* cooperate in immortalized human Schwann cells in culture, endowing these cells with oncogenic properties such as increased proliferation and anchorage-independent growth (Figure 5). This further emphasizes the importance of these two distinct signaling pathways in the tumorigenesis of both NF1-associated and sporadic MPNSTs. More intense drug development to find novel drug targets for these two signaling pathways should be addressed. Importantly, we now have rapid GEMMs that recapitulate both the human NF1-associated [9] and sporadic MPNSTs for preclinical testing of therapeutic agents.

## Supplementary Material

Supplementary Figure 1: shows the histological and immunohistochemical analyses of peripheral nervous system phenotype in *Dhh*-Cre; *Pten_flox/flox_* (*ΔPten*) animals. The relatively low numbers of Ki67-positive cells detectable in peripheral nervous tissue sections of *ΔPten* animals indicate a low-grade peripheral nerve sheath tumor (PNST) phenotype. As expected, pAkt levels were much higher in the peripheral nervous tissue sections of *ΔPten* animals when compared with wild-type FVB/N (FVB) control animals. pErk levels in all peripheral nervous tissue sections were comparable between *ΔPten* and FVB animals. All peripheral nervous tissue sections taken from *ΔPten* and FVB animals were positive for Olig2 staining, consistent with nerve association.
Supplementary Figure 2: demonstrates the high-grade PNSTs that develop in our mouse model recapitulate human sporadic malignant peripheral nerve sheath tumors (MPNSTs). Using high power view of hematoxylin-eosin (HE) stained PNSTs, key phenotypic features seen in human MPNSTs were also present in tumors taken from our mouse model. These include hypercellularity, haphazard cell arrangement, poor cell differentiation, nuclear pleomorphism and nuclear hyperchromasia. In addition, PNSTs taken from our mouse model were highly reactive for Ki67, indicating high mitotic activity, similar to human high-grade tumors.

## Figures and Tables

**Table 1:** Occurrence of different peripheral nervous system phenotype detected in various experimental and control cohorts.

Genotype	*N*	Median survival age (days)	*n*	Enlarged DRG (mean ± SD)	Tumor grade	BP	TN	SN	LP
*Pt* *en* ^*f*/*f*^; *Cnp*-*EGFR *	13	26	12	13.7 ± 7.8	High	100%	92%	50%	50%
*Pt* *en* ^*f*/*f*^	12	247	10	6.7 ± 3.8	Low	100%	90%	80%	10%
*Pt* *en* ^*f*/+^; *Cnp*-*EGFR *	11	415	5	5.8 ± 2.6	Hyperplasia/Low	100%	100%	80%	20%

All mice were transgenic for *Dhh*-Cre. *f/f*: *flox/flox*; *f/+*: *flox/+*; *N*: total number of mice in each cohort; Median: median survival age; *n*: number of mice examined for the occurrence of various peripheral nervous system phenotype; DRG: number of enlarged dorsal root ganglia isolated (mean ± standard deviation); Grade: tumor grade was determined by histological evaluation as described in Section 2. High: high-grade PNST; Low: low-grade PNST; Hyperplasia/Low: hyperplasia to low-grade PNST. Percentage of animals in each cohort that displayed the following peripheral nervous system phenotype; BP: enlarged brachial plexi; TN: enlarged trigeminal nerves; SN: enlarged sciatic nerves; LP: enlarged sacral plexi.
