# Supplementary material for: Conditional Inactivation of Pten with EGFR Overexpression in Schwann Cells Models Sporadic MPNST
Source: Sarcoma. 2012 Dec 18;2012:620834. doi: 10.1155/2012/620834 (PMC3539440; doi:10.1155/2012/620834)
Supplement: Supplementary file 1 — Supplementary Figure 1: shows the histological and immunohistochemical analyses of peripheral nervous system phenotype in Dhh-Cre; Ptenflox/flox (ΔPten) animals. The relatively low numbers of Ki67-positive cells detectable in peripheral nervous tissue sections of ΔPten animals indicate a low-grade peripheral nerve sheath tumor (PNST) phenotype. As expected, pAkt levels were much higher in the peripheral nervous tissue sections of ΔPten animals when compared with wild-type FVB/N (FVB) control animals. pErk levels in all peripheral nervous tissue sections were comparable between ΔPten and FVB animals. All peripheral nervous tissue sections taken from ΔPten and FVB animals were positive for Olig2 staining, consistent with nerve association. Supplementary Figure 2: demonstrates the high-grade PNSTs that develop in our mouse model recapitulate human sporadic malignant peripheral nerve sheath tumors (MPNSTs). Using high power view of hematoxylin-eosin (HE) stained PNSTs, key phenotypic features seen in human MPNSTs were also present in tumors taken from our mouse model. These include hypercellularity, haphazard cell arrangement, poor cell differentiation, nuclear pleomorphism and nuclear hyperchromasia. In addition, PNSTs taken from our mouse model were highly reactive for Ki67, indicating high mitotic activity, similar to human high-grade tumors. [file 620834.f1.pdf]

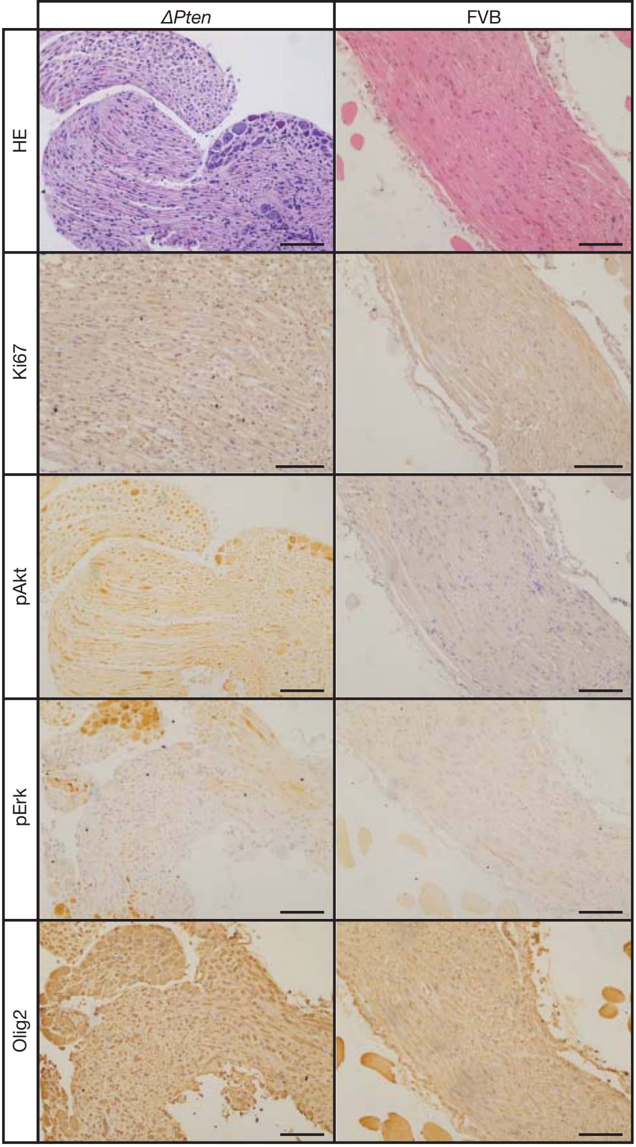

Supplementary Figure 1 Histological and immunohistochemical (IHC) analyses of peripheral nervous system phenotype. Standard hematoxylin-eosin staining (HE) was performed on all peripheral nervous system tissue sections. IHC staining using antibodies against the proliferative marker (Ki67), activated *Pi3k*/*Akt* signaling by phospho-Akt (pAkt), activated *Ras*/*Mapk*/*Erk* signaling by phospho-Erk1/2 (pErk) and the Schwann cell/oligodendrocyte lineage marker (Olig2). Representative peripheral nervous system tissues taken from *Dhh*-Cre; *Pten*$^{flox/flox}$ (Δ*Pten*) and wild-type FVB/N (FVB) animals. Scale bars, 100 µm.
