# Supplementary material for: Conditional Inactivation of Pten with EGFR Overexpression in Schwann Cells Models Sporadic MPNST
Source: Sarcoma. 2012 Dec 18;2012:620834. doi: 10.1155/2012/620834 (PMC3539440; doi:10.1155/2012/620834)
Supplement: Supplementary file 2 [file 620834.f2.pdf]

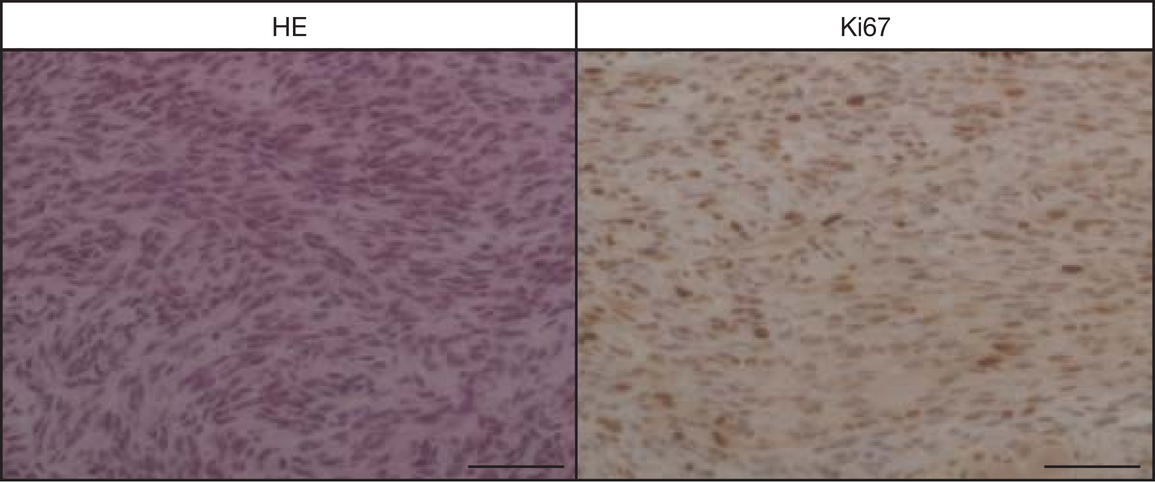

Supplementary Figure 2 High-grade PNSTs that develop in our mouse model recapitulate human sporadic MPNSTs. **Left**, high power view of tumor stained with HE shows features that are typical of MPNST in humans, including hypercellularity, haphazard cell arrangement, poor cell differentiation, variable nuclear size and shape (nuclear pleomorphism), and nuclei that stain very dark (nuclear hyperchromasia). **Right**, virtually all cells in this high power field show reactivity with Ki67, marker for nuclear mitotic activity, similar to human high-grade tumors. Scale bars, 50 μm.
